# Revisiting an overlooked biomarker: CSF-IgA demonstrates significant diagnostic value for viral encephalitis in a case-control study

**DOI:** 10.3389/fcimb.2026.1882946

**Published:** 2026-09-01

**Authors:** Dongxing Wang, Jiaping Xu, Bo Lv, Rui Wang, Shuangxiao Deng, Yanlin Zhang, Rong Zou, Yu Wang, Baochun Zhou, Jie Pan, Yongjun Cao

**Affiliations:** 1 Department of Neurology, The Second Affiliated Hospital of Soochow University, Suzhou, China; 2 Department of Pharmacy, The Second Affiliated Hospital of Soochow University, Suzhou, China; 3 Department of Intensive Care Medicine, Liuyang People’s Hospital, Changsha, Hunan, China; 4 Information and Big Data Center, The Second Affiliated Hospital of Soochow University, Suzhou, China; 5 Department of Emergency and Critical Care Medicine, The Second Affiliated Hospital of Soochow University, Suzhou, China

**Keywords:** cerebrospinal fluid, diagnostic model, immunoglobulin A, prediction, viral encephalitis

## Abstract

**Background:**

Viral encephalitis (VE) is a serious central nervous system infection, yet its diagnosis is challenged by non-specific clinical manifestations, low pathogen detection rates, and often normal routine cerebrospinal fluid (CSF) findings. The diagnostic potential of CSF immunoglobulin A (CSF-IgA) has been largely overlooked. This study aimed to evaluate the diagnostic value of CSF-IgA for VE.

**Methods:**

This single-center, retrospective case-control study analyzed data from 233 patients with VE and 80 symptomatic controls, collected over a 15.5-year period (January 1, 2010 to June 30 2025). A multivariable logistic regression model was constructed using variables that were significant in univariate analysis (p < 0.05). Model performance was rigorously evaluated in the overall cohort for discrimination (area under the receiver operating characteristic curve, AUC), calibration (using calibration plots and the Brier score), and clinical utility (via decision curve analysis). The model was further validated in a predefined subgroup of VE patients (n=50) with normal routine CSF findings, defined as normal CSF pressure, cell count, total protein, albumin, glucose, and chloride levels.

**Results:**

The final parsimonious diagnostic model retained three robust predictors: CSF-IgA (OR 2.56, 95% CI: 1.73–4.06), CSF white blood cell count (OR 1.45, 95% CI: 1.25–1.76), and serum albumin (OR 0.81, 95% CI: 0.71–0.91). It demonstrated excellent discrimination in the overall cohort (AUC 0.933, 95% CI: 0.913–0.955), was well-calibrated (Brier score = 0.091), and provided superior net benefit across a wide threshold probability range. Importantly, robust performance was maintained in the clinically challenging subgroup with normal routine CSF parameters (AUC 0.812, 95% CI: 0.730–0.873; Brier score = 0.171).

**Conclusions:**

CSF-IgA may serve as a promising adjunctive biomarker for viral encephalitis. A diagnostic model incorporating CSF-IgA showed good performance, even in atypical cases with normal routine CSF profiles, indicating its potential utility as an auxiliary tool for early VE diagnosis. However, given the single-center retrospective design, prospective external validation is warranted before routine clinical implementation.

## Background

Viral encephalitis is an inflammatory condition of the central nervous system caused by viral infection, characterized by fever, prodromal symptoms, altered mental status, seizures, and focal neurological deficits (Yong et al., 2023; Siciliano et al., 2022). It has a global incidence of 1.4-13.8 per 100,000 person-years, with viral pathogens, particularly herpesviruses (Duerlund et al., 2025; Aksamit, 2021), accounting for approximately 70% of confirmed infectious encephalitis cases (Tyler, 2018; Said and Kang, 2025). This incidence varies somewhat with patient age and immune status. The World Health Organization (WHO), in its 2025 technical brief (Jesudason, 2025), reported over 80,000 deaths globally in 2021, long-term sequelae in 50% of survivors, and an annual disease burden approaching 5 million disability-adjusted life years, underscoring its substantial public health impact (Habis et al., 2025b; Wang et al., 2022). Although the 2025 WHO guidelines emphasize rapid diagnosis as essential for effective management (WHO, 2025), implementing this in clinical practice remains challenging.

Current diagnosis of viral encephalitis primarily relies on clinical manifestations, physical signs, electroencephalography (EEG), routine cerebrospinal fluid (CSF) analysis, and neuroimaging. However, only about 76.3% of patients present with abnormal routine CSF findings (Habis et al., 2025a; Bloch and Glaser, 2025), and 30%–62% of cases lack an identified pathogen (Sonneville et al., 2025; Yong et al., 2023), largely due to suboptimal test sensitivity, delayed specimen collection, and low viral loads in CSF. Although advances in CSF viral PCR (Sanbonmatsu-Gámez et al., 2021) and metagenomic next-generation sequencing (mNGS) (Wilson et al., 2019; Bloch et al., 2023) have improved diagnostic capabilities, their clinical adoption remains limited due to high costs, lengthy turnaround times, and interference from background nucleic acids (Wilson and Tyler, 2022; Chen et al., 2024). These constraints highlight the urgent need for novel biomarkers that are cost-effective, rapid, and reliable (The Lancet Neurology, 2025).

Although immunoglobulin A (IgA) is primarily known for its role in mucosal immunity, emerging evidence suggests its involvement in CNS inflammatory responses (Fitzpatrick et al., 2020; Isho et al., 2021). Elevated CSF IgA levels may reflect altered blood-brain barrier permeability and immune activity at meningeal border structures, including dura-associated lymphoid tissue (DALT) and skull bone marrow channels (Smyth and Kipnis, 2025; Rustenhoven and Kipnis, 2022; Wheeler and Quintana, 2025). These structures facilitate the homing of IgA^+^ plasma cells to the dural venous sinuses and drive intrathecal immunoglobulin synthesis (Desestret et al., 2015; Ayroza Galvão Ribeiro Gomes et al., 2023), potentially activating antigen-presenting cells to initiate adaptive immune responses. Despite this biological plausibility, the diagnostic value of CSF IgA for viral encephalitis has long been overlooked, and a comprehensive assessment is lacking. Therefore, employing a case-control design, this study aimed to comprehensively evaluate the diagnostic performance of CSF IgA for viral encephalitis—including in patients with normal routine CSF findings—and to provide an evidence base for its development as a practical and cost-effective biomarker, in response to the WHO’s call for improved diagnostic solutions (2025, Matthews et al., 2025).

## Methods

### Study design and participants

#### Study design

This single-center, retrospective case-control study adhered to a pre-defined protocol for participant selection to minimize bias. Candidate cases for the viral encephalitis group were independently screened by two experienced physicians. A parallel, blinded screening process was implemented for the symptomatic control group by two deputy chief physicians unaware of the study grouping. For both groups, concordant selections were included, and any discordant cases underwent blinded adjudication by a third senior physician, with a final consensus reached by an expert panel. All statistical analyses, encompassing pre-specified primary and subgroup analyses, were conducted by a PhD statistician with over five years of clinical research experience, who remained blinded to group allocation throughout the process to safeguard against analysis bias.

#### Study subjects

##### Viral encephalitis group

Inclusion Criteria (Patients must meet all of the following):

1. Diagnosis: Fulfilled the diagnostic criteria for encephalitis proposed by the International Encephalitis Consortium (Venkatesan et al., 2013):

- Major Criterion (Required): Altered mental status (including decreased or altered level of consciousness, lethargy, or personality change) lasting ≥24 hours, with no identifiable alternative cause.
- Minor Criteria (≥3 required):
  - Fever (Axillary temperature ≥38 °C/100.4°F) within 72 hours before or after presentation.
  - New-onset generalized or partial seizures.
  - New-onset focal neurological signs.
  - CSF white blood cell count ≥5/mm³. (In cases with significant CSF red blood cells, e.g., from atraumatic tap, the following correction formula could be applied: True CSF WBC = actual CSF WBC – [(Blood WBC × CSF RBC)/Blood RBC]).
  - Neuroimaging (MRI or CT) demonstrating new-onset or acute parenchymal abnormalities consistent with encephalitis.
  - Electroencephalography (EEG) abnormalities indicative of encephalitis, not attributable to other causes.

2. Age: Between 18 and 80 years, inclusive.

3. Symptom Onset to Lumbar Puncture: The interval from symptom onset to lumbar puncture was ≤ 2 weeks.

4. Data Completeness: Availability of complete clinical data, including CSF analysis (pressure, routine biochemistry, and immunoglobulins IgA, IgG, IgM), EEG, neuroimaging, and comprehensive blood tests (complete blood count, liver/kidney function, electrolytes, glucose, thyroid function, viral serology, autoimmune antibodies, and tumor markers).

Exclusion Criteria (Patients were excluded if they met any of the following):.

1. CNS Disorders: Coexisting CNS diseases such as autoimmune encephalitis, multiple sclerosis, neuromyelitis optica spectrum disorders, CNS vasculitis, neurosarcoidosis, Parkinson’s disease, Alzheimer’s disease, spinocerebellar ataxia, other neurodegenerative diseases, or a history of severe head trauma or cerebrovascular disease.

2. Major Systemic Diseases:

- Systemic autoimmune diseases: systemic lupus erythematosus, rheumatoid arthritis, Sjögren’s syndrome).
- Immunodeficiency disorders:(defined as a known history of HIV/AIDS, active use of immunosuppressive therapy (e.g., prednisone ≥20 mg/day for >2 weeks, chemotherapy, or biologic agents) within the past 3 months, or a history of organ transplantation).
- Diabetes mellitus.
- Thyroid disease.
- Abnormal liver function(ALT or AST greater than 2 times the upper limit of normal (ULN), or total bilirubin greater than 1.5 times ULN, or the presence of decompensated cirrhosis).
- Abnormal renal function (defined as an estimated glomerular filtration rate (eGFR) < 60 mL/min/1.73 m², a serum creatinine level above the upper limit of normal).
- History of hematological malignancies or other organ malignancies.

3. Other Infections or Inflammatory States:

- Concurrent or suspected intracranial infections other than viral meningoencephalitis (including bacterial, fungal, tuberculous, syphilitic, or parasitic infections).
- History of other active systemic infections (e.g., pneumonia, urinary tract infection, gastrointestinal infection) within 4 weeks prior to enrollment.
- History of hepatitis virus (HAV, HBV, HCV, HDV, HEV), or syphilis infection.

4. Prior Treatment: Administration of corticosteroids, intravenous immunoglobulin before the lumbar puncture during this hospitalization.

5. Special Physiological Status: Pregnancy or lactation.

6. Substance Use: History of chronic alcohol dependence or drug abuse/addiction.

7. Comorbid chronic nasal, oral, or skin diseases.

##### Symptomatic control group

Due to ethical constraints prohibiting the acquisition of CSF from healthy volunteers, symptomatic controls were selected from the medical records database of the Second Affiliated Hospital of Soochow University. These were patients who underwent lumbar puncture for non-specific neurological symptoms (e.g., dizziness, neuralgia, or insomnia) but were ultimately diagnosed with non-inflammatory, non-organic conditions. The symptomatic controls were matched to the viral encephalitis cases based on age and sex.

Inclusion Criteria (Controls must meet all of the following):

1. Clinical Presentation: Underwent lumbar puncture for symptoms such as dizziness, neuralgia, or insomnia.

2. Age: Between 18 and 80 years, inclusive.

3. Final Diagnosis: Discharge diagnosis belonging to the following non-inflammatory, non-organic disorders: tension-type headache, primary insomnia, benign paroxysmal positional vertigo, or peripheral vestibular vertigo.

4. Normal CSF Parameters:

- Pressure: 80–180 mmH_2_O.
- WBC count: ≤ 5/mm³.
- RBC count: 0.
- Total protein: 150–450 mg/L.
- Albumin: 0–350 mg/L.
- Normal glucose: 2.5 – 4.5 mmol/L.
- CSF Chloride: 120–132 mmol/L).
- Negative fungal smear and culture.
- Negative bacterial culture, viral nucleic acid test, and mNGS (if tested).

5. Normal Blood Test Results:

- Normal liver and kidney function (defined as having ALT, AST, total bilirubin, and albumin levels within the institutional reference range, and an estimated glomerular filtration rate (eGFR) ≥ 90 mL/min/1.73m²).
- Normal fasting blood glucose, serum glucose level < 6.1 mmol/L.
- Normal electrolytes and thyroid function (defined as serum sodium, potassium, chloride, calcium, TSH, FT3 and FT4 levels all within the normal reference ranges of our institutional laboratory).
- Negative tumor markers, viral serology (HAV, HBV, HCV, HDV, HEV, HIV), syphilis, and autoantibodies.
- WBC count: 3.5–9.5 × 10^9^/L.
- C-reactive protein (CRP) < 8 mg/L.
- Procalcitonin (PCT) < 0.05 ng/mL.

6. Normal Imaging and Electrophysiology: No significant abnormalities on brain MRI/CT, EEG, or chest CT.

7. Data Completeness: Availability of complete clinical data, identical to the requirements for the viral encephalitis group.

Exclusion Criteria (Controls were excluded if they met any of the following):

1. History of neurodegenerative or neuroinflammatory diseases (e.g., multiple sclerosis, neuromyelitis optica spectrum disorders, CNS vasculitis, neurosarcoidosis, Parkinson’s disease, Alzheimer’s disease, spinocerebellar ataxia).
2. History of cerebrovascular disease, cranial or spinal structural injury, epilepsy, or status epilepticus.
3. History of confirmed or suspected central nervous system infection.
4. History of confirmed or suspected Guillain-Barré syndrome.
5. Diagnosis of systemic autoimmune disease (e.g., systemic lupus erythematosus, Sjögren’s syndrome, rheumatoid arthritis).
6. Active systemic infection or fever (Axillary temperature >37.3 °C) within 4 weeks prior to enrollment.
7. History of intracranial tumors, leptomeningeal carcinomatosis, paraneoplastic syndromes, or other malignancies.
8. Immunodeficiency disorders or long-term use of immunosuppressants/corticosteroids.
9. History of hepatitis virus (HAV, HBV, HCV, HDV, HEV), HIV, or syphilis infection.
10. Familial hereditary diseases.
11. Pre-existing diabetes mellitus or thyroid disease.
12. Pregnancy or lactation.
13. History of alcohol or drug abuse/addiction.
14. Comorbid chronic nasal, oral, or skin diseases.

Both the control and case groups employed rigorous exclusion criteria, with the purpose of minimizing, to the greatest extent possible, the confounding influences on CSF IgA levels.

### Participant screening and data collection

Investigators screened the electronic medical records of the Second Affiliated Hospital of Soochow University according to the pre-defined inclusion and exclusion criteria from January 2010 to June 2025. Cases identified during initial screening were cross-checked, and only those achieving consensus were finally included. The following data were collected from all enrolled participants in both case and control groups: sex, age, clinical symptoms, physical signs, medical history, electroencephalography (EEG), and neuroimaging (MRI) findings. Laboratory measures included blood test results—complete blood count (white blood cell, absolute lymphocyte, absolute neutrophil, and absolute monocyte counts), total protein, and albumin—and cerebrospinal fluid (CSF) parameters, including opening pressure, cell count, total protein, albumin, and quantitative immunoglobulin levels (IgA, IgG, and IgM).

### CSF-IgA measurement

Cerebrospinal fluid immunoglobulin A (CSF-IgA) levels were quantified in the central laboratory of the Second Affiliated Hospital of Soochow University. Following lumbar puncture, 2 mL of CSF was centrifuged at 3000 rpm for 10 minutes, and the resulting supernatant was analyzed. Measurements were performed using an immunoturbidimetric assay on a Beckman Coulter IMMAGE 800 analyzer with the Beckman Coulter Low Concentration Immunoglobulin A Test Kit. The laboratory reference interval for CSF-IgA was 0–2 mg/L. The analytical platform, methodology, and CSF-IgA reference interval remained unchanged throughout the entire study period (January 1, 2010 to June 30, 2025). The laboratory adhered to rigorous internal quality control protocols and participated in external proficiency testing throughout the study period to ensure the reliability and consistency of all measurements.

### Statistical analysis

Statistical analyses were performed using R software (version 4.2.2). Continuous variables were tested for normality using the Shapiro-Wilk test. Non-normally distributed data are presented as median (interquartile range) and were compared using the Mann-Whitney U test. Categorical variables are summarized as number (percentage) and were compared using the chi-square test.

To identify independent predictors of viral encephalitis, we first conducted univariate logistic regression analyses for all candidate variables. Variables with a significant association in univariate analyses (P < 0.05) and clinical relevance were considered for inclusion in the multivariable logistic regression model. The results from these regression analyses are expressed as odds ratios (OR) with 95% confidence intervals (CI). The final diagnostic prediction model was refined to include only those predictors that provided stable and statistically significant contributions, aiming for parsimony and clinical applicability.

The model’s performance was evaluated in terms of discrimination, calibration, and clinical utility. Discrimination was assessed by the area under the receiver operating characteristic (ROC) curve (AUC), calibration was evaluated using calibration plots, and clinical utility was examined via decision curve analysis (DCA).

To assess the diagnostic value of CSF-IgA in atypical presentations, the model was validated in a predefined subgroup of viral encephalitis patients with normal routine CSF parameters, testing its generalizability in this clinically challenging population. All statistical tests were two-sided, and a P-value < 0.05 was considered statistically significant.

## Results

**Figure 1:**
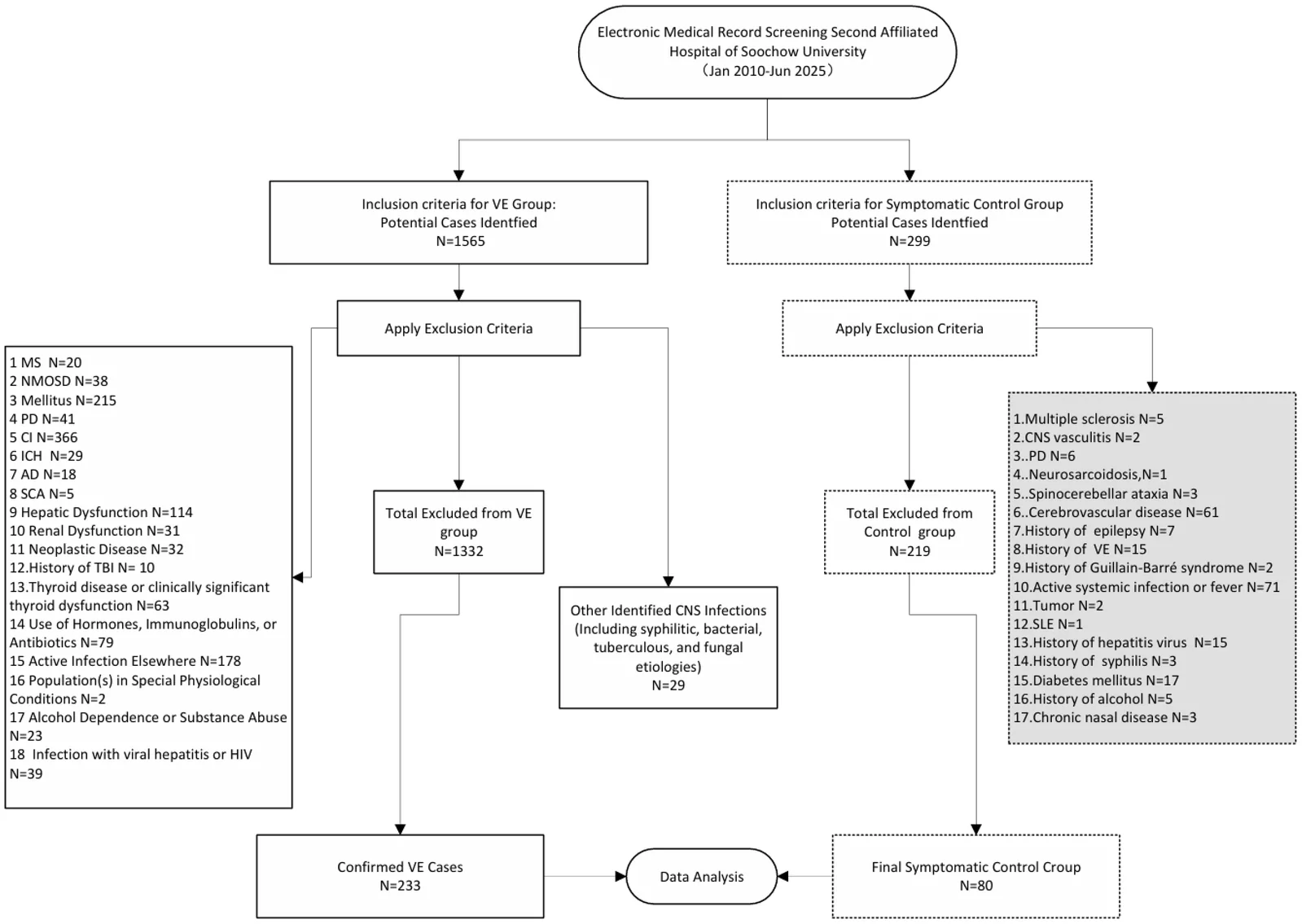
Flowchart of participant enrollment (STROBE diagram). AD, Alzheimer’s disease; CI, Cerebral Infarction; ICH, Intracerebral Hemorrhage; MS, Multiple Sclerosis; NMOSD, Neuromyelitis optica spectrum disorder; PD, Parkinson’s disease; SA, Spinocerebellar ataxia; SLE, Systemic lupus erythematosus; TBI, Traumatic brain injury; VE, Viral encephalitis.

We initially identified 1,565 patients meeting the preliminary inclusion criteria for viral encephalitis (VE) through a systematic screening of electronic medical records at the Second Affiliated Hospital of Soochow University between January 1, 2010, and June 30, 2025. After applying all pre-specified exclusion criteria, 1,332 patients were excluded, resulting in a final study cohort of 233 confirmed VE cases. Using the same screening procedures, 80 Symptom control subjects were selected from the same source population and matched to the cases based on age and sex. The complete screening and enrollment process is detailed in **Figure 1** (a flowchart designed in accordance with STROBE guidelines) (von Elm et al., 2007).

### Baseline characteristics of the study population

This study included a total of 313 participants, comprising 233 patients in the viral encephalitis (VE) group and 80 individuals in the symptomatic control group. The VE group consisted of 118 males and 115 females, with a median age of 36 years (IQR: 27.0–52.0). The control group included 39 males and 41 females, with a median age of 37.5 years (IQR: 32.0–54.0).

Among the 233 VE patients, 172 exhibited abnormal electroencephalography (EEG) findings, characterized by diffuse or focal moderate- to high-amplitude theta and delta waves, with occasional sharp or spike wave discharges. Additionally, 32 patients showed abnormal signals on brain MRI, involving regions such as the temporal lobe, parietal lobe, and cerebellum.

Among the 233 patients in the case group, 179 (76.8%) underwent mNGS of CSF using a shotgun approach. Viral nucleic acids were detected in 72 (40.2%) of these patients, accounting for a total of 74 viral detections (two patients had co-infections). The distribution of viral detections was as follows: herpes simplex virus (n=26), varicella-zoster virus (n=19), Epstein–Barr virus (n=9), human polyomavirus (n=6), human herpesvirus types 6 and 7 (n=5 in total; 3 of HHV-6 and 2 of HHV-7), SARS-CoV-2 (n=4), torque teno virus (n=2), rhinovirus (n=1), enterovirus (n=1), and cytomegalovirus (n=1). Co-infection was observed in two patients: one had simultaneous detection of human polyomavirus and herpes simplex virus, and the other had simultaneous detection of herpes simplex virus and Epstein–Barr virus. In the control group of 80 patients, 54 underwent CSF mNGS testing, and all tested negative for viruses.

Comparison of baseline characteristics between the two groups revealed no significant differences in age, sex, serum globulin, or peripheral blood absolute lymphocyte count (*p* > 0.05). In contrast, the VE group demonstrated significantly higher values in multiple cerebrospinal fluid (CSF) parameters (*p* < 0.05). Specifically, CSF levels of IgA, IgG, IgM, total protein, albumin, and white blood cell count were all markedly elevated in the VE group (*p* < 0.001). Furthermore, the VE group had lower serum albumin levels and peripheral white blood cell counts (*p* < 0.001), while absolute neutrophil count, absolute monocyte count, and CSF pressure were moderately higher than those in the control group (*p* < 0.05). Detailed results are presented in **Table 1**.

**Table 1:** Baseline characteristics of the study population.

Variable	Control group (n=80)	VE group (n=233)	P-value
Sex, Female, n (%)	41(51.30)	115 (49.30)	0.871
Age, years	37.50 (32.00, 54.00)	36.00 (27.00, 52.00)	0.169
CSF parameters			
IgA, mg/L	1.54 (1.24, 2.31)	3.99 (2.35, 7.52)	< 0.001
IgG, mg/L	22.5 (17.88, 28.50)	33.70 (23.50, 56.6)	< 0.001
IgM, mg/L	0.36 (0.23, 0.58)	0.75 (0.39, 1.90)	< 0.001
TP, mg/L	321.00 (253.00, 361.00)	466.00 (310.00, 684.00)	< 0.001
Alb, mg/L	174.15 (129.98, 225.00)	273.00 (167.90, 453.01)	< 0.001
WBC, ×10^6^/L	1.00 (0, 2.25)	8.00 (2.00, 50.00)	< 0.001
Pressure, mmH_2_O	145.00 (120.00, 160.00)	150.00 (120.00, 190.00)	0.045
Blood parameters			
Alb, g/L	44.10 (42.58, 46.05)	42.00 (39.10, 44.25)	< 0.001
Glob, g/L	23.80 (21.90, 27.12)	25.20 (22.70, 27.70)	0.130
WBC, ×10^9^/L	5.57 (4.80, 6.75)	4.84 (4.35, 5.90)	< 0.001
ALC, ×10^9^/L	1.80 (1.40, 2.20)	1.60 (1.20, 2.10)	0.053
ANC, ×10^9^/L	3.40 (3.05, 4.73)	4.20 (3,00 5.80)	0.028
AMC, ×10^9^/L	0.40 (0.30, 0.50)	0.50 (0.30, 0.60)	0.012

Data are presented as n (%) or median (interquartile range). Alb, albumin; ALC, absolute lymphocyte count; AMC, absolute monocyte count; ANC, absolute neutrophil count; CSF, cerebrospinal fluid; Glob, globulin; Ig, immunoglobulin; TP, total protein; WBC, white blood cell count.

### Univariate and multivariate logistic regression analyses

Univariate logistic regression analysis identified several factors significantly associated with viral encephalitis (p < 0.05), including CSF-IgA, CSF-IgG, CSF-IgM, CSF total protein, CSF albumin, CSF white blood cell (WBC) count, CSF pressure, serum albumin, blood WBC count, and absolute neutrophil count.

Multivariate logistic regression analysis, which adjusted for potential confounders, revealed four independent predictors: CSF-IgA (OR = 3.39, 95% CI: 2.07–5.94, p < 0.001), CSF total protein (OR = 1.01, 95% CI: 1.00–1.02, p = 0.049), CSF WBC count (OR = 1.57, 95% CI: 1.31–1.97, p < 0.001), and serum albumin (OR = 0.82, 95% CI: 0.72–0.92, p = 0.001).

Among these independent predictors, CSF-IgA demonstrated a strong and stable association with viral encephalitis, with an odds ratio of 3.39 (95% CI: 2.07–5.94), indicating it is a robust independent risk factor. CSF WBC count also showed a notable positive association (OR = 1.57), while serum albumin was independently and inversely associated with VE (OR = 0.82). Detailed results are presented in **Table 2**.

**Table 2:** Univariate and multivariate logistic regression analysis for predictors of viral encephalitis.

Variable	Univariate analysis OR (95% CI)	P-value	Multivariate analysis OR (95% CI)	P-value
Sex,	1.08 [0.65, 1.80]	0.770		
Age	0.99 [0.98, 1.01]	0.343		
CSF-IgA	3.09 [2.23, 4.53]	<0.001	3.39 [2.07 5.94]	< 0.001
CSF-IgG	1.07 [1.05, 1.10]	<0.001	0.96 [0.89, 1.03]	0.175
CSF-IgM	4.55 [2.50, 9.46]	<0.001	0.66 [0.66, 1.88]	0.119
CSF-TP	1.01 [1.00, 1.01]	<0.001	1.01 [1.00, 1.02]	0.049
CSF-Alb	1.01 [1.00, 1.01]	<0.001	0.99 [0.98, 1.00]	0.094
CSF-WBC	1.50 [1.29, 1.79]	<0.001	1.57 [1.31, 1.97]	< 0.001
CSF-Pressure	1.01 [1.00, 1.01]	0.011	1.01 [1.00, 1.02]	0.117
Serum-Alb_	0.82 [0.75, 0.89]	<0.001	0.82 [0.72, 0.92]	0.001
Serum-Glob	1.04 [0.98, 1.11]	0.223		
Blood-WBC	0.83 [0.72, 0.94]	0.004	0.86 [0.64, 1.13]	0.286
Blood-ALC	1.02 [0.94, 1.24]	0.764		
Blood-ANC	1.14 [1.02, 1.30]	0.032	0.89 [0.68, 1.19]	0.410

### Predictive model development and evaluation

**Figure 2:**
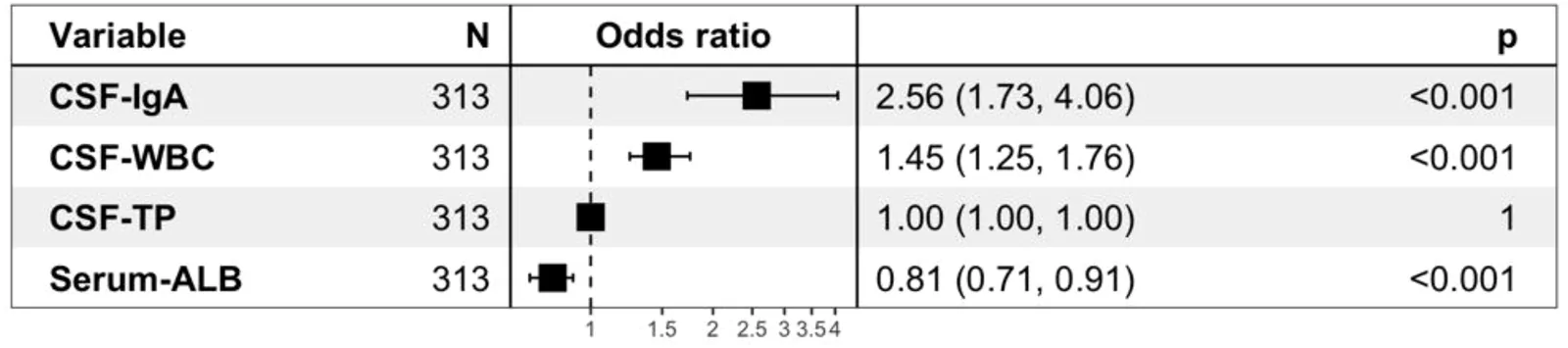
Forest plot of the final multivariable logistic regression model for viral encephalitis prediction. ALB, albumin; CSF, cerebrospinal fluid; Ig, immunoglobulin; WBC, white blood cell count.

From the initial multivariable analysis (**Table 2**), we developed a parsimonious clinical prediction model by retaining only the predictors that sustained strong statistical significance and clinical relevance. The final model incorporated CSF-IgA, CSF-WBC count, and serum albumin. The effect sizes of these predictors in the final model, as visualized in the forest plot (**Figure 2**), were CSF-IgA (OR 2.56, 95% CI: 1.73–4.06), CSF-WBC (OR 1.45, 95% CI: 1.25–1.76), and serum albumin (OR 0.81, 95% CI: 0.71–0.91). Cerebrospinal fluid total protein (CSF-TP), despite its initial significance, was excluded from the final model as its association was not stable and its inclusion did not meaningfully improve model performance. Following model development, we assessed its apparent performance and conducted internal validation on the full cohort to estimate generalizability.

**Figure 3:**
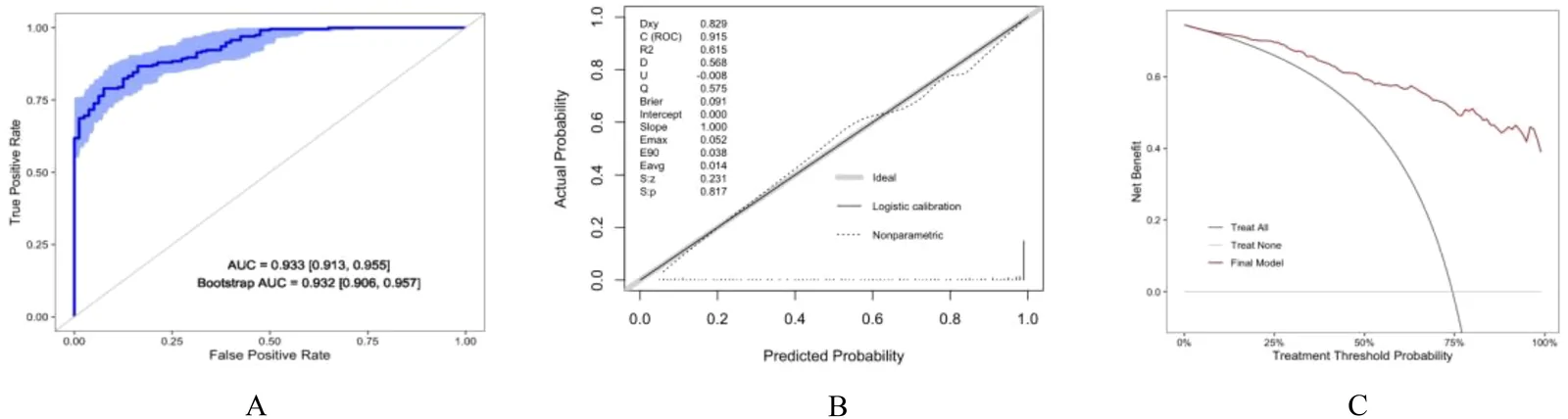
Performance evaluation of the viral encephalitis prediction model. **(A)** Receiver operating characteristic (ROC) curve of the prediction model. The model shows an area under the curve (AUC) of 0.933 (95% CI: 0.913 - 0.955). Internal validation using bootstrap resampling (n=2000) confirmed the robust performance (bootstrap AUC = 0.932, 95% CI: 0.906 - 0.957). **(B)** Calibration plot of the predictive model. The solid line depicts the logistic calibration of the model, while the dashed line represents the ideal reference. Key calibration metrics, including a Brier score of 0.091, a calibration slope of 1.000, an intercept of 0.000, and a maximum estimation error (Emax) of 0.052, and an average absolute error (Eavg) of 0.014, collectively demonstrate a strong agreement between predicted probabilities and observed outcomes. **(C)** Decision curve analysis of the prediction model. The plot compares the net benefit of using the model to guide intervention against the default strategies of treating all or no patients. The model demonstrates superior clinical utility across a wide range of clinically reasonable threshold probabilities, as indicated by its higher net benefit.

As shown in **Figure 3**, the model exhibited strong overall performance in discrimination, calibration, and clinical utility. The ROC analysis (**Figure 3A**) demonstrated excellent diagnostic accuracy, with an AUC of 0.933 (95% CI: 0.913–0.955). Bootstrap internal validation (n = 2000) confirmed robust discriminative ability (AUC = 0.932, 95% CI: 0.906–0.957). The calibration plot (**Figure 3B**) indicated close agreement between predicted and observed outcomes, supported by a Brier score of 0.091, a calibration slope of 1.000, and minimal estimation errors (Emax = 0.052, Eavg = 0.014). Decision curve analysis (**Figure 3C**) further confirmed the model’s clinical value, showing a consistent net benefit across a wide range of threshold probabilities compared to default strategies.

### Subgroup analysis in patients with normal CSF parameters

To assess the model’s performance in patients with diagnostically challenging, atypical viral encephalitis (VE), we conducted a subgroup analysis of 50 VE patients presenting with normal routine cerebrospinal fluid (CSF) parameters, including pressure (80–180 mmH_2_O), cell count (WBC count: ≤5/mm³), total protein (150–450 mg/L), albumin (0–350 mg/L), glucose (2.5 – 4.5 mmol/L), chloride levels (120–132 mmol/L) and negative fungal smear and culture. The baseline characteristics of this subgroup are summarized in **Table 3**.

**Table 3:** Baseline characteristics of viral encephalitis patients stratified by cerebrospinal fluid profile.

Variables	Abnormal CSF profile (n = 183)	Normal CSF profile (n = 50)	p
Sex, Female, n(%)	82 (45.00)	34 (66.67)	0.013
Age, years	37.00 (28.00, 53.50)	33.50 (25.00, 46.75)	0.109
CSF parameters			
IgA, mg/L	4.97 (2.72, 9.15)	2.37 (1.70, 3.07)	< 0.001
IgG, mg/L	40.90 (26.80, 70.50)	21.35 (17.70, 26.70)	< 0.001
IgM, mg/L	1.13 (0.48, 2.50)	0.41 (0.25, 0.62)	< 0.001
TP, mg/L	520.00 (397.50, 797.00)	290.00 (233.50, 337.50)	< 0.001
Alb, mg/L	349.90 (216.85, 516.45)	152.25 (123.70, 187.42)	< 0.001
WBC, ×10^6^/L	14.00 (4.00, 85.50)	2.00 (1.00, 2.75)	< 0.001
Pressure, mmH_2_O	160.00 (120.00, 200.00)	140.00 (112.50, 158.75)	< 0.001
Blood parameters			
Alb, g/L	41.90 (39.05, 44.30)	42.25 (40.40, 43.50)	0.790
Glob, g/L	25.30 (22.90, 28.45)	24.90 (21.80, 26.70)	0.074
WBC, ×10^9^/L	4.73 (4.30, 5.80)	5.35 (4.62, 6.10)	0.046
ALC, ×10^9^/L	1.60 (1.10, 2.10)	1.65 (1.40, 2.18)	0.184
ANC, ×10^9^/L	4.60 (3.00, 6.30)	3.35 (2.62, 4.30)	< 0.001
AMC, ×10^9^/L	0.50 (0.30, 0.60)	0.40 (0.30, 0.60)	0.429

Data are presented as n (%) or median (interquartile range). Patients were stratified into “Abnormal CSF profile” (≥1 abnormality in CSF pressure, total protein, albumin, or WBC count) and “Normal CSF profile” (all parameters within normal range).

CSF, cerebrospinal fluid; Ig, immunoglobulin; TP, total protein; Alb, albumin; Glob, globulin; WBC, white blood cell count; ALC, absolute lymphocyte count; ANC, absolute neutrophil count; AMC, absolute monocyte count.

**Figure 4:**
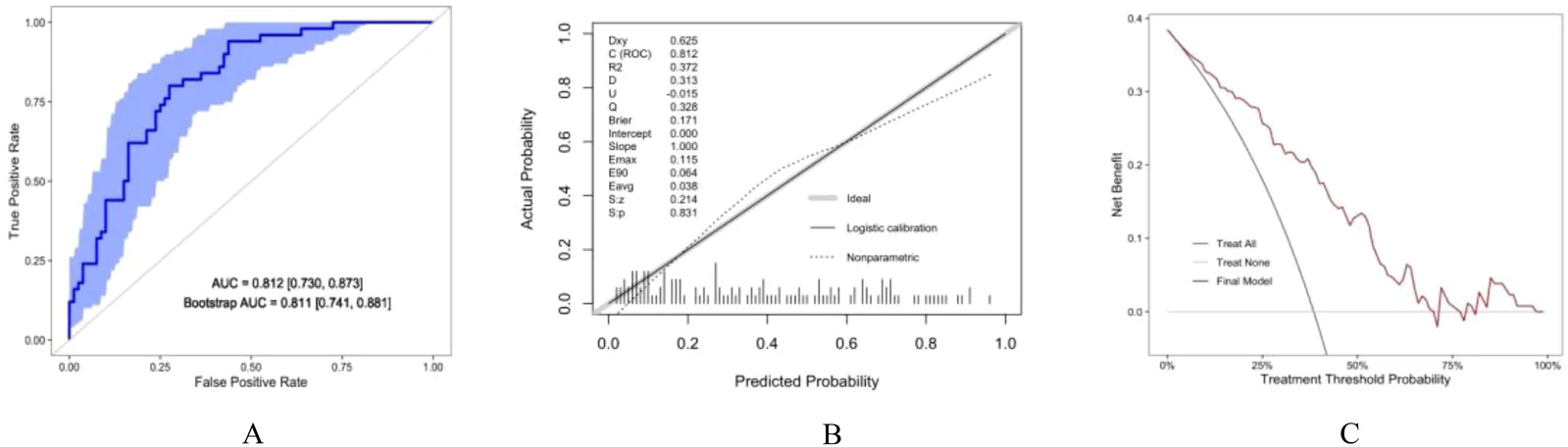
Performance evaluation of the prediction model in the viral encephalitis subgroup. **(A)** Receiver operating characteristic (ROC) curve of the prediction model in the viral encephalitis subgroup. The model demonstrates strong discriminative ability in this validation subset, with an area under the curve (AUC) of 0.812 (95% CI: 0.730 - 0.873). Internal validation via bootstrap resampling (n=2000) confirmed the robustness of this estimate (bootstrap AUC = 0.811, 95% CI: 0.741 - 0.881). **(B)** Calibration plot of the prediction model in the viral encephalitis subgroup. The solid line represents the logistic calibration of the model, and the dashed line represents the ideal reference. Key calibration metrics include a Brier score of 0.171, a calibration slope of 1.000, and an intercept of 0.000. The maximum estimation error (Emax) was 0.115 and the average absolute error (Eavg) was 0.038, indicating good agreement between predicted and observed outcomes in this patient subset. **(C)** Decision curve analysis of the prediction model in the viral encephalitis subgroup. In the viral encephalitis subgroup, the model demonstrated sustained clinical utility, providing a higher net benefit than the strategies of treating all or no patients across a wide threshold probability range (approximately 2% to 75%).

As shown in **Figure 4**, the model maintained robust performance in this clinically challenging population. The receiver operating characteristic (ROC) curve (**Figure 4A**) demonstrated strong discriminatory power, with an area under the curve (AUC) of 0.812 (95% CI: 0.730-0.873). Internal validation using bootstrap resampling (n=2000) confirmed the reliability of this estimate (bootstrap AUC = 0.811, 95% CI: 0.741-0.881). Calibration performance (**Figure 4B**) remained satisfactory, with a Brier score of 0.171, calibration slope of 1.000, and minimal estimation errors (Emax = 0.115, Eavg = 0.038). Decision curve analysis (**Figure 4C**) further demonstrated the model’s clinical utility, showing superior net benefit across a wide threshold probability range (approximately 2% to 75%) compared to default clinical strategies.

These results confirm that the prediction model maintains clinically useful diagnostic performance even in the challenging subgroup of VE patients with normal routine CSF findings.

## Discussion

This case-control study identified cerebrospinal fluid immunoglobulin A (CSF-IgA) as a strong and independent diagnostic biomarker for viral encephalitis (VE). Our findings confirm that CSF-IgA levels are significantly elevated in VE patients. More importantly, after adjusting for multiple potential confounders—including routine CSF parameters (white blood cell count and total protein) and other immunoglobulins—in the multivariate regression analysis, CSF-IgA retained a significant and stable association with VE (adjusted OR = 3.39, 95% CI: 2.07–5.94, p < 0.001), underscoring its independent diagnostic value.

The main findings and implications of this study are as follows.

First, the multivariate diagnostic model developed in this study demonstrated excellent overall performance. By integrating the key independent predictors—CSF-IgA, CSF-WBC count, and serum albumin—the model achieved a high area under the curve (AUC = 0.933) in the derivation cohort. Furthermore, both the calibration plot and decision curve analysis confirmed its accurate risk prediction capability and favorable clinical net benefit across a wide threshold probability range. This indicates that combining multiple biomarkers can provide more robust diagnostic performance than any single indicator alone.

Second, a notable strength of this study is the successful validation of the diagnostic model in a challenging subgroup of VE patients who presented with completely normal routine CSF findings. A substantial proportion of VE patients can exhibit normal routine CSF parameters (e.g., WBC count, total protein, glucose, and chloride), making clinical diagnosis particularly difficult and prone to missed diagnoses or delayed treatment (Chen et al., 2022). This clinically important phenomenon, however, is not well recognized in routine practice (Bloch and Glaser, 2025). As highlighted by Habis et al. in a large cohort study involving nearly 600 adult patients with encephalitis, more than a quarter of patients with infectious encephalitis lacked CSF pleocytosis, including almost a quarter of those with herpes simplex virus type 1 (HSV-1) encephalitis (Habis et al., 2025a). These findings underscore that the absence of CSF cellular reaction does not rule out viral infection of the brain parenchyma, as encephalitis may occur without concomitant meningeal inflammation (Bloch and Glaser, 2025). In our study, a total of 50 VE patients (21.45% of the case group) presented with completely normal routine CSF profiles—including pressure, WBC count, total protein, glucose, and chloride—a proportion slightly lower than that reported in the Habis study (19.0–25.3% depending on etiology) (Habis et al., 2025a). Importantly, even within this diagnostically elusive subgroup, our CSF-IgA-based diagnostic model maintained good discriminative ability (AUC = 0.812). This strongly suggests that CSF-IgA measurement can compensate for the limitations of conventional CSF analysis, providing crucial laboratory evidence for diagnosing the most challenging VE patients who would otherwise be missed by routine testing. In line with the editorial commentary by Glaser and Bloch (Glaser and Bloch, 2009), our findings support the aggressive diagnostic evaluation of all patients with suspected encephalitis, regardless of whether they meet traditional criteria for CSF pleocytosis, and highlight the potential role of novel biomarkers such as CSF-IgA in this context.

Third, in our multivariate model, CSF-IgA demonstrated stronger independent predictive power than CSF-IgG or CSF-IgM. This finding directly supports that it may play a unique role in the neuroimmunological pathogenesis of viral encephalitis (VE) and highlights its potential as a specific biomarker at the neuroimmune interface. To our knowledge, this is the first study to directly compare the diagnostic performance of the three immunoglobulin isotypes in VE using a case-control design. Future mechanistic studies are warranted to elucidate whether this superiority is driven by isotype-specific receptor interactions or compartmentalized adaptive immune regulation.

In the same multivariate model, serum albumin was independently and inversely associated with VE (OR = 0.82, 95% CI: 0.72–0.92, p < 0.001). This finding is consistent with previous literature and further strengthens the clinical relevance of our model (Wiedermann, 2021). The possible mechanisms may involve VE-related immunoinflammatory responses that suppress hepatic albumin synthesis, as well as albumin leakage into the CSF following blood–brain barrier disruption. In the context of central nervous system infections, hypoalbuminemia often reflects an increased systemic inflammatory burden, which may contribute to the observed association (Mantovani and Garlanda, 2023), However, given the observational nature of our study, these mechanistic interpretations remain speculative, residual confounding cannot be fully excluded, and the exact mechanisms warrant further investigation.

From a clinical perspective, VE diagnosis requires integrating symptoms, signs, EEG findings, neuroimaging, and history. Pathogen identification is important, but viral nucleic acid results in immunocompetent patients must be interpreted with caution—positivity does not confirm etiology without full clinical correlation. Although mNGS and multiplex PCR are highly sensitive and specific, they are limited by cost, equipment, accessibility, turnaround time, background interference, and interpretation difficulties. Among the detected viruses in our study, some are clinically significant while others remain uncertain, and pathogen detection is only part of the diagnostic workup. Therefore, CSF-IgA, a routine, inexpensive, and rapid test, offers a complementary tool when molecular testing is unavailable or delayed. Nevertheless, these practical advantages do not diminish the need to critically appraise the methodological limitations of our study.

This study has certain limitations, First, due to ethical constraints, CSF from healthy volunteers was not available. We therefore used a “symptomatic control” group comprising patients with non-specific neurological symptoms (e.g., tension-type headache, primary insomnia, benign paroxysmal positional vertigo, or peripheral vestibular vertigo) who were ultimately diagnosed without organic or inflammatory CNS diseases. Although we implemented stringent inclusion and exclusion criteria to ensure that controls had normal CSF and blood parameters and no definitive pathological conditions, this design may still introduce potential bias. The non-specific symptoms in these controls might stem from unknown, subtle physiological or pathological disturbances, potentially creating minor differences from a truly “healthy” state. However, this control selection strategy is widely adopted in biomarker studies within neurology (Cohen et al., 2022). Importantly, using this “non-specific symptom” control group might lead to an underestimation, rather than an overestimation, of the true diagnostic performance of CSF-IgA, as it increases the difficulty of distinguishing VE from other non-inflammatory conditions that cause similar complaints. Therefore, the diagnostic value of CSF-IgA reported in this study might be a conservative estimate, and its performance in differentiating VE from truly healthy individuals could potentially be higher. Second, the VE group had a median age of 36 years (IQR 27.0–52.0), and our findings may not be generalizable to older adult populations, in whom the epidemiology and etiology of VE differ substantially. Third, due to the retrospective design, paired serum samples were not available for all patients, precluding direct comparison between CSF and serum IgA levels and limiting our ability to distinguish intrathecal synthesis from blood-brain barrier disruption. Fourth, we excluded immunocompromised individuals (e.g., those with HIV, long-term immunosuppressant use, or organ transplantation) to maintain cohort homogeneity; consequently, our results cannot be generalized to this high-risk population. Fifth, we did not include a disease control group with autoimmune encephalitis or other neuroinflammatory disorders, which represent the main differential diagnoses of VE; therefore, the specificity of CSF-IgA for VE in this clinically challenging context remains to be established. Sixth, the sample size within individual pathogen subgroups was limited, precluding meaningful subgroup analysis to determine whether CSF-IgA levels vary by specific viral etiology. Finally, as a single-center retrospective study, selection bias and residual confounding cannot be excluded; external validation in prospective cohorts with age-stratified, pathogen-specific, and disease-control designs is warranted to confirm the generalizability of our findings.

## Conclusion

Our findings suggest that CSF-IgA may represent a promising candidate adjunctive biomarker for viral encephalitis. A predictive model incorporating CSF-IgA demonstrated good discriminatory performance and favorable clinical utility. Notably, CSF-IgA may provide diagnostic complementarity in challenging cases with normal routine CSF findings. Therefore, we suggest that clinicians and laboratories consider the potential value of CSF-IgA as an adjunctive tool in the diagnostic workup of viral encephalitis, which may assist in earlier identification and risk stratification. However, given the single-center retrospective design and internal validation only, prospective external validation is warranted before routine clinical implementation.

## Data Availability

The original contributions presented in the study are included in the article/supplementary material. Further inquiries can be directed to the corresponding authors.
